# Initial Clinical Experience of MR-Guided Radiotherapy for Non-Small Cell Lung Cancer

**DOI:** 10.3389/fonc.2021.617681

**Published:** 2021-03-10

**Authors:** Cathryn B. Crockett, Pamela Samson, Robert Chuter, Michael Dubec, Corinne Faivre-Finn, Olga L. Green, Sara L. Hackett, Fiona McDonald, Clifford Robinson, Anna-Maria Shiarli, Michael W. Straza, Joost J. C. Verhoeff, Maria Werner-Wasik, Gregory Vlacich, David Cobben

**Affiliations:** ^1^ Radiotherapy Related Research, The Christie NHS Foundation Trust, Manchester, United Kingdom; ^2^ Department of Radiation Oncology, Washington University in St. Louis, St. Louis, MO, United States; ^3^ Division of Cancer Sciences, The University of Manchester, Manchester, United Kingdom; ^4^ Department of Radiation Oncology, University Medical Center Utrecht, Utrecht, Netherlands; ^5^ Department of Radiotherapy, Royal Marsden NHS Foundation Trust, London, United Kingdom; ^6^ Department of Radiotherapy, Cambridge University Hospitals NHS Foundation Trust, Cambridge, United Kingdom; ^7^ Department of Radiation Oncology, Froedtert and the Medical College of Wisconsin, Milwaukee, WI, United States; ^8^ Department of Radiation Oncology, Sidney Kimmel Cancer Center at Thomas Jefferson University, Philadelphia, PA, United States

**Keywords:** magnetic resonance imaging (MRI), external beam radiotherapy, adaptive, image-guided radiotherapy (IGRT), MR-guided radiotherapy (MRgRT), stereotactic body radiation therapy (SBRT), non-small cell lung cancer (NSCLC)

## Abstract

Curative-intent radiotherapy plays an integral role in the treatment of lung cancer and therefore improving its therapeutic index is vital. MR guided radiotherapy (MRgRT) systems are the latest technological advance which may help with achieving this aim. The majority of MRgRT treatments delivered to date have been stereotactic body radiation therapy (SBRT) based and include the treatment of (ultra-) central tumors. However, there is a move to also implement MRgRT as curative-intent treatment for patients with inoperable locally advanced NSCLC. This paper presents the initial clinical experience of using the two commercially available systems to date: the ViewRay MRIdian and Elekta Unity. The challenges and potential solutions associated with MRgRT in lung cancer will also be highlighted.

## Introduction

### Lung Cancer in Context

SBRT plays an important role in the curative-intent treatment of medically inoperable patients with early-stage NSCLC (1, 2). Radical radiotherapy, either alone or in combination with concurrent chemotherapy (followed by adjuvant immunotherapy in eligible patients), is the curative-intent treatment option open to those with locally advanced disease (1, 2). It is therefore crucial to plan and deliver the radiotherapy using technologies that can fully optimise the therapeutic index. This can be achieved with strategies that increase the probability of tumor control, while simultaneously reducing the probability of normal tissue complications (3).

Intra-fractional anatomical changes, attributed to cardiac and respiratory motion, pose the greatest challenge for accurate radiotherapy delivery (4–6).

These changes could lead to under-dosage of the tumor and over-dosage of the organs at risk (OARs), which could lead to an increased risk of recurrence or long term toxicity (6–8). Therefore, there is a clinical need to ensure that the tumor is receiving the prescribed dose while the dose to the OARs is kept to a minimum, e.g., to reduce cardiac toxicity and its related sequelae (7–9). MRgRT has the potential to facilitate this.

### The Role of MRgRT in Lung Cancer

**Figure 1:**
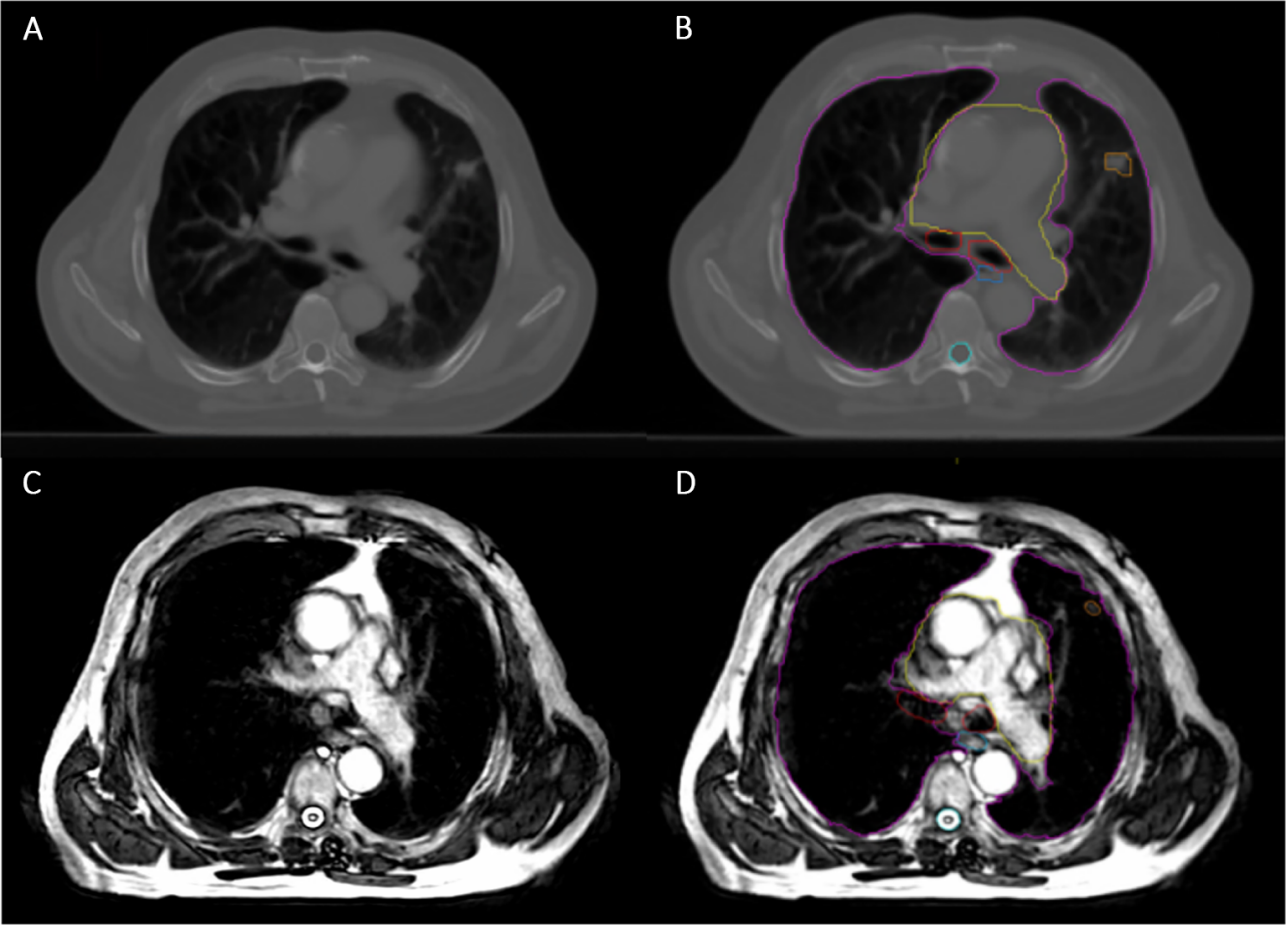
Planning Computed Tomography (CT) image compared with MR image on the Unity. **(A)** Planning CT image showing small peripheral right lung tumor. **(B)** The same planning CT image including tumor and OAR contours (pink = lungs, yellow = heart, red = proximal bronchial tree, blue = oesophagus, cyan = spinal cord and orange = Gross Tumor Volume). **(C)** Unity MR image for the same patient, using 3D Vane – balanced Turbo Field Echo (bTFE) sequence. **(D)** The same Unity MR image including tumor and OAR contours, as described before.

MRgRT has a number of potential benefits which could be exploited in the lung cancer setting. The excellent soft tissue contrast of MRI may result in the improved delineation of challenging target volumes, such as those located centrally or close to and/or invading adjacent structures, and OARs (**Figure 1**) (10). MRgRT may also enable the potential for daily plan adaptation and margin reduction, which could lead to improved OAR dose sparing (11, 12). Daily plan adaptation could account for anatomical and physiological changes throughout the course of radiotherapy and thereby has the potential to improve dosimetric accuracy (12). The “beam-on” capabilities of MRgRT systems permit real-time monitoring during radiotherapy treatment. This may allow for motion mitigation by gating or tracking and therefore again may facilitate the use of smaller margins (12). MRgRT may therefore improve the therapeutic index of radiotherapy treatment for lung cancer. Another advantage of MRgRT is the ability to acquire functional imaging to assess response and to potentially permit adaptive workflows based on biological information (13).

Ongoing research should help to highlight the specific groups of lung cancer patients most likely to benefit from MRgRT. Daily adaptive SBRT continues to be investigated as an option for (ultra-) central early-stage disease (14–19). MRgRT may also prove advantageous to patients with locally advanced disease, especially in more challenging cases where other imaging modalities, e.g., CT (Computed Tomography) and 18-Fluorodeoxyglucose-Positron Emission Tomography (FDG-PET) may fail to provide enough planning information. Examples of this include the ability to better assess tumor invasion into surrounding tissue (e.g., mediastinum, chest-wall) or where the tumor is abutting collapsed lung. Isotoxic dose escalation may be another option in this patient cohort (20). Finally, oligometastatic lung cancer patients may benefit from improved target definition and treatment accuracy, particularly for sites of disease within the abdomen (21).

**Figure 2:**
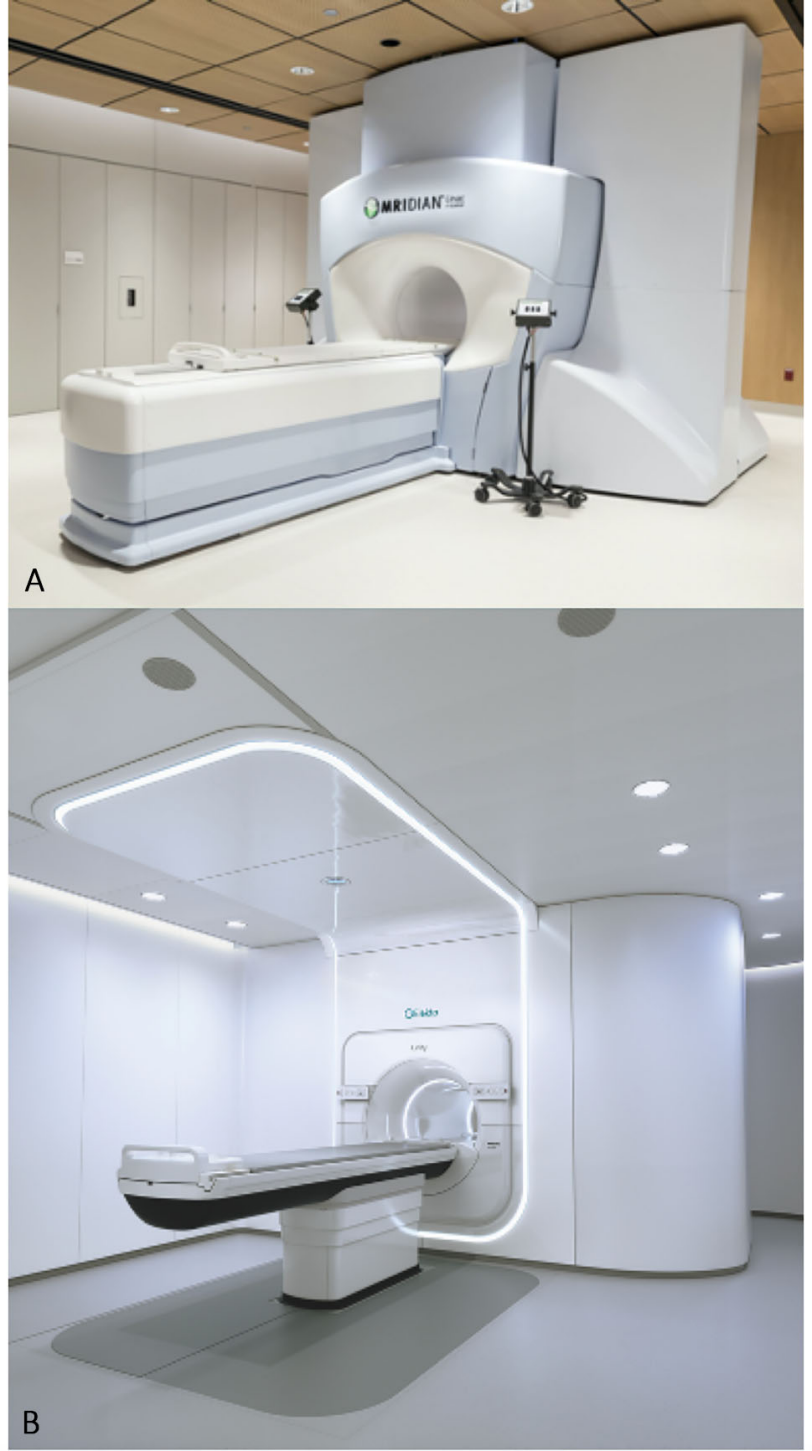
The two commercially available MR-guided radiotherapy systems. **(A)** The MRIdian (ViewRay Inc, USA). **(B)** The Unity (Elekta, Sweden).

There are currently five different MR-radiotherapy delivery systems documented in the literature but to our knowledge, only two of these are in clinical use (22, 23). This paper will focus on the commercially available MRIdian (ViewRay Inc, USA) and Unity (Elekta, Sweden) systems, and their use in the lung cancer setting (**Figure 2**).

### The MRIdian System

The first commercially available system, the MRIdian, was Food and Drug Administration (FDA) approved in 2012 and then introduced clinically in 2014. Initially, it consisted of a three-headed cobalt source system with a low field magnet (0.35 T) (24). The second version, which replaced the three-headed cobalt source with a 6 megavoltage (MV) linear accelerator, was FDA approved in February 2017 and the first patient was subsequently treated in July 2017 (24). There are now 34 MRIdian systems in 13 countries across the globe and to date over 10,000 cancer patients have been treated and more than 95 peer-reviewed articles have been published (25). ViewRay has also established a multicentre Clinical Co-operative Think Tank (C^2^T^2^) which is a collaborative group comprising clinical MRIdian users from over 20 international institutions. Its role is to enable the sharing of clinical data and best practice as well as ongoing research and evaluation of MRgRT.

### The Unity System

The Unity is the second commercially available system with a magnetic field strength of 1.5 T and a 7 MV linear accelerator (24). An international consortium, including teams from seven research centers from across the United Kingdom, Europe, and the United States, was set up in 2012 to facilitate the collaborative investigation of the system and its introduction into clinical practice (26). The first patient was treated on the Unity machine in Utrecht in May 2017, as part of a cohort of patients with spinal metastases (27). The system received FDA approval in December 2018. Currently, there are 16 Unity systems in 11 countries across the globe and to date; more than 1,000 patients have been treated (28). As of March 2020, 236 peer-reviewed publications on the development and implementation of the system have been produced (28, 29).

## Methodology

A literature search was performed on PubMed to identify relevant published literature, including abstracts. It was performed initially in May 2020 but updated in October 2020. The search terms used were: (“MR-guided” OR “magnetic resonance-guided” OR MRI-guided OR “magnetic resonance imaging-guided” OR MR-Linac) AND (“non-small cell lung cancer” OR NSCLC OR “lung cancer” OR thorax OR thoracic OR lung) AND (radiotherapy OR “radiation therapy” OR SBRT OR SABR OR “adaptive radiotherapy” OR “adaptive radiation therapy” OR “image-guided radiotherapy” OR “image-guided radiation therapy” OR stereotactic). Identified articles were reviewed manually and cross-checked for other relevant papers.

## Initial Clinical Experiences

### Background

The initial clinical experience of thoracic MRgRT has mainly included the use of SBRT for the treatment of early-stage lung cancer (30–38). Owing to concerns relating to bronchial toxicity, SBRT use was initially restricted to those with tumors >2 cm from the central airways (15, 39). However, in recent years an increasing number of publications have shown that dose-adapted SBRT regimens can be delivered in centrally located tumors (14, 19). However, severe toxicities have been reported, particularly in patients with ultra-central tumors and prospective studies are needed in this setting (19).

MRgRT with its superior soft tissue contrast and potentially improved and adaptive planning and treatment delivery accuracy may help to reduce uncertainties and enable a reduction in planning margins and volumes (12). This in turn increases the scope for safer treatment of (ultra-) central tumors. In addition, the reduction in planning margins could make conventionally fractionated radiotherapy more attractive for patients with locally advanced lung cancer, minimising the risk of radiation pneumonitis and/or acute oesophagitis.

### *In Silico* Studies With the MRIdian

The potential clinical advantage of MRgRT for intrathoracic disease was initially explored for SBRT of (ultra-) central tumors. A retrospective *in silico* analysis of ultra-central thoracic and abdominal malignancies demonstrated that initial treatment plans violated OAR constraints approximately 63% of the time when applied to subsequent daily fraction MR imaging (21). Online adaptive treatments (re-planning to account for anatomical changes) could have resolved all violations (21). Subsequent *in silico* retrospective analysis of hypofractionated MRgRT (12 fractions) for (ultra-) central tumors suggested a similar benefit with this approach (16).

### Clinical Experience With the MRIdian

This system was first introduced clinically in 2014 and within the initial phase, 61 patients with intra-thoracic tumors were treated (30). The feasibility of MRgRT with daily online adaptive treatment for SBRT of ultra-central thoracic tumors was subsequently evaluated in a prospective Phase I study (17). Five patients were included and all received 50 Gy in five fractions. Adaptive treatments (to account for anatomical changes) were required for four out of five patients and in ten out of 25 delivered fractions. Seventy percent of the adaptive re-plans were carried out for OAR violations and 30% to improve PTV coverage. Local disease control was 100% at 6 months, with no grade 3 or higher toxicities. While patients included in this study and the two retrospective *in silico* studies had both NSCLC and oligometastatic disease from a non-lung primary, there does not appear to be any significant difference with regard to the potential benefit of adaptive MRgRT by histology (16, 21).

Other institutions have had similar clinical experiences using MRgRT to treat lung tumors (primary or oligometastases from non-lung primaries), but reports of clinical outcomes as a whole remain lacking for NSCLC (31–33, 35, 36). Adaptive MRgRT for lung SBRT was found to improve OAR sparing in 88% of treatments and improve PTV coverage compared to a non-adaptive plan in a small cohort (34). Daily adaptive MR-guided SBRT for central lung lesions was also found to improve PTV coverage in 61% of fractions with a reduction in the number of OAR violations (18).

More recently, the use of MRgRT to deliver lung SBRT in a single fraction, under real-time image guidance, has been reported (37). Re-optimised plans following on-table adaptation showed improved PTV coverage to 95% compared with 89.8% in predicted plans. Stereotactic magnetic resonance-guided adaptive radiation therapy (SMART) has also been used to treat high-risk lung cancer cases (central tumors, re-irradiation and patients with interstitial lung disease) (38). Improvements in PTV coverage were highlighted alongside low rates of toxicity and encouraging early clinical outcomes. In general, the clinical consequences of improvements in PTV coverage and OAR sparing have not been extensively reported, however.

A prospective Phase I-II trial (ClinicalTrials.gov ID NCT04115254) is currently open. It aims to evaluate the feasibility and efficacy of SMART in patients with lung, pancreatic, and renal cancer. Another institutional single-arm Phase II study with safety lead-in (ClinialTrials.gov ID: NCT03916419) is open and exploring the role of MR-guided radiotherapy in the definitive management of inoperable, locally advanced NSCLC. They are assessing the feasibility and clinical benefit of MRgRT in hypofractionated (60 Gy in 15 fractions) concurrent chemoradiotherapy and consolidation with Durvalumab is being examined.

### *In Silico* Studies With the Unity

A study assessing the feasibility of treating nine early-stage lung cancer patients with SBRT found that clinically acceptable lung SBRT plans were possible (40). Small differences in dose to the target and OARs (especially increased dose to skin) were noted with MRgRT, but with minimal clinical impact expected. This was also found in patients with locally advanced NSCLC (20). Furthermore, the improved imaging capabilities meant that PTV margin reduction was possible, in turn facilitating increased OAR sparing and isotoxic dose escalation. A subsequent study of five patients assessed the effects of density overrides on treatment planning for MRgRT in lung cancer (41). The team concluded that when using density overrides, recalculation of optimised plans using the original CT is essential, to avoid under-dosage of the tumor.

### Clinical Experience With the Unity

The Multiple Outcome Evaluation of Radiation Therapy Using the MR-Linac (MOMENTUM) Study (ClinicalTrials.gov ID: NCT04075305) has been open since February 2019. It is a prospective, multi-institutional, international cohort study/registry investigating the implementation of the Unity MR-Linac and its ongoing development. All patients treated on the MR-linac are eligible for inclusion in MOMENTUM across 12 disease sites, including lung cancer (42). The objective of MOMENTUM is to collect and evaluate technical and clinical data to allow for optimisation of software with the ultimate aim of improving local disease control, patient survival, and quality of life.

At the time of writing this paper, the Medical College of Wisconsin (MCW) has treated one patient with intrathoracic disease (inoperable stage III NSCLC) with concurrent chemoradiotherapy at a dose of 60 Gy in 30 fractions. Their radiotherapy was delivered using the Adapt To Position (ATP, virtual couch shift) workflow and was well tolerated (43).

At University Medical Center Utrecht (UMCU), 10 patients with (ultra-) central tumors have been treated thus far at a dose of 60Gy in 8 to 12 fractions. All patients were treated by daily generating a new treatment plan that was optimised to the daily anatomy visualized on the 3D MR Dataset, using an ATP and Adapt To Shape (ATS, adapted to anatomical changes) workflow (43). Treatments have been well tolerated by patients. In addition to MOMENTUM registration for MR-linac treatments, all lung cancer patients are prospectively registered in the Utrecht Cohort for Lung cancer Outcome Reporting and trial inclusion (U-COLOR). Its “Trials-within-Cohorts” (TwiCs) design enables efficient, fast, and pragmatic testing of new interventions in a randomised fashion (44).

Finally, a team in Shandong, China have treated one patient with SBRT for stage I NSCLC at a dose of 56Gy in seven fractions, with an ATP workflow applied to all fractions. Treatment was well tolerated and a follow-up CT, one-month post-treatment, showed a good local response.

**Table 1** summarizes the clinical experience, to date.

**Table 1:** Clinical experience to date, by stage.

Disease stage	Team	Machine	No. of patients	Tumor location	Fractionation schedule	Sequence used	Immobilization/positioning	Adaption	Gating/tracking	Couch time (min)
I/II	Thomas et al. 2018 (32)	MRIdian	5	Peripheral and central	50–54Gy/3–4#	TrueFISP	NR	NR	Tracking	>20
		Cobalt-60								
	Padgett et al. 2018 (34)	MRIdian	3 (1 primary lung)	Peripheral	50Gy/5#	NR	NR	To anatomy	NR	NR
		Cobalt-60								
	De Costa et al. 2018 (Abstract) (35)	MRIdian Cobalt-60	14 (11 primary lung)	NR	40–50Gy/5#	NR	NR	NR	Both	NR
	Henke et al., 2018 (17)	MRIdian Cobalt-60	5 (1 primary lung)	Ultra-central	50Gy/5#	NR	NR	To anatomy	Gating	Median = 69
	Finazzi et al. 2019 (36)	MRIdian Cobalt-60 or MR-Linac	23 (25 tumors - 14 primary lung)	Peripheral	54–60Gy/3–8#	TrueFISP	NR	To anatomy	Gating	Median from changing room to end of delivery:
										Cobalt-60 = 62
										MR Linac = 48
	Finazzi et al. 2020 (37)	MRIdian MR-Linac	10 (8 primary lung)	Peripheral	34Gy/1#	TrueFISP	NR	To anatomy	Both	Median from changing room to end of delivery: 120
	Finazzi et al. 2020 (38)	MRIdian Cobalt-60 or MR-Linac	50 (29 primary lung)	Peripheral and central	54Gy–60Gy/3–12#	TrueFISP	NR	To anatomy	Both	Median from changing room to end of delivery:
										Cobalt-60 = 60
										MR-Linac = 49
	Li et al., 2019 (Poster, 14^th^ Elekta MR-Linac Consortium meeting)	Unity	1	Peripheral	56Gy/7#	T2 3D	Custom vacuum bag	ATP	Intermittent “motion monitoring”	<30
	Merckel et al., 2020 (Private correspondance)	Unity	10	Central/ultra-central	60Gy/8–12#	T2 3D	Mattress, arms down	ATS	Nil	Median = 39
III	Straza et al., 2019 (Private correspondance)	Unity	1	Peripheral and central	60Gy/30#	4D Vane TFE	Vac fix, arms up	ATP	“Real-time monitoring”	30–35
IV	Padgett et al. 2018 (34)	MRIdian Colbalt-60	3 (2 oligo-metastases)	Peripheral and central	48–50Gy/4#	NR	NR	To anatomy	NR	NR
	De Costa et al. 2018 (Abstract) (35)	MRIdian Cobalt-60	14 (3 oligo-metastases)	NR	40–50Gy/5#	NR	NR	NR	Both	NR
	Henke et al. 2019 (17)	MRIdian Cobalt-60	5 (4 oligo-metastases)	Ultra-central	50Gy/5#	NR	NR	To anatomy	Gating	Median = 69
	Finazzi et al. 2019 (36)	MRIdian Cobalt-60 or MR-Linac	23 (25 tumors - 11 oligometastases)	Peripheral	54–60Gy/3–8#	NR	NR	To anatomy	Gating	Median from changing room to end of delivery:
										Cobalt-60 = 62
										MR Linac = 48
	Finazzi et al. 2020 (37)	MRIdian MR-Linac	10 (2 oligo-metastases)	Peripheral	34Gy/1#	TrueFISP	NR	To anatomy	Both	Median from changing room to end of delivery = 120
	Finazzi et al. 2020 (38)	MRIdian Cobalt-60 or MR-Linac	50 (21 oligo-metastases)	Peripheral and central	54Gy–60Gy/3–12#	TrueFISP	NR	To anatomy	Both	Median from changing room to end of delivery:
										Cobalt-60 = 60
										MR-Linac = 49

An effort was made to include only the most recent data to avoid duplicate reporting of patients. NR, not recorded; ATP, Adapt To Position; ATS, Adapt To Shape; TFE, turbo field echo; TrueFISP, True Fast Imaging with Steady Precession.

## Challenges

The integration of MRI into radiotherapy planning and delivery systems has led to the need for changes in the radiotherapy workflow (43, 45). These changes relate to the potential for daily online imaging, plan adaptation, and re-optimisation while ensuring patients are comfortable on the treatment couch. Such workflows are still in development. The ultimate goal is to have an “MR-only” radiotherapy workflow (46). This concept incorporates MRI diagnostic scans, MRI use for target delineation (“planning MRI”), treatment monitoring and real-time adaption, and finally the use of functional MR sequences during treatment to assess for early response and enable adaptation as necessary (13, 46, 47).

Despite its potential benefits, the implementation of MRgRT into routine clinical practice has proven challenging for reasons including cost-effectiveness, patient selection, departmental logistics, changes to workflow, and technical challenges (12, 22, 48).

### Cost-Effectiveness

A number of surveys on the implementation of MRgRT have indicated that health economics and/or accessibility may be the main reasons behind its slow uptake (22, 48). MRgRT systems are expensive and the delivery of value-based healthcare has been acknowledged as a global priority (48, 49). Given their expense it will be important to carefully define indications for their clinical use.

### Patient Selection

Once a clinical program has been established, and the demand exceeds the MR-Linac capacity, identifying patients that will benefit most from MRgRT is crucial (48). At Washington University, a bi-weekly triage meeting has been established to review proposed treatments and help determine if and when MRgRT is appropriate based on clinical indicators and machine availability.

### Departmental Logistics (Including Training)

The delivery of MRgRT requires input from a multidisciplinary team comprising physicians, radiographers, and physicists. Therefore it depends upon adequate staff resourcing, logistical co-ordination, and appropriate training (12, 23, 50). Access to multidisciplinary contour training with MRI (e.g., workshops) for staff is limited. MR contouring recommendations for GTV and OARs along with multidisciplinary training, in conjunction with a radiologist, are essential to ensure reproducibility of delineation (51, 52). MR-specific GTV and OAR contouring recommendations are currently in development.

### Workflow

The use of daily plan adaptation inevitably leads to a longer clinical workflow time (23, 43, 45). As a result, the number of patients treated daily on an MR-Linac is much more limited compared to a standard linac. Overall fraction time can be further extended if the time between initial image capture and plan acceptance is too long. This is due to an increased risk of intra-fractional movement which may result in the plan no longer being acceptable for treatment (48). An increase in couch time in combination with the smaller bore size of the MR-Linac due to the presence of MR coils can lead to difficulty with patient positioning and potential patient-comfort related issues with claustrophobia, noise, feeling cold, paraesthesia, and anxiety (12, 45, 53, 54).

There are multiple steps in the process where optimisation can be implemented to reduce treatment time or improve accuracy and reproducibility of adaptive planning. One option includes the use of specialized MRgRT radiographers appropriately trained in OAR contouring to improve efficiency (12, 50). Another option may be to use auto-segmentation of OARs and even target volumes (55). Nevertheless, it is still early in the clinical implementation of MRgRT to know which interventions are most effective, so this remains an ongoing area of investigation.

### Technical Challenges

#### MR Imaging

Obtaining high-quality MR images for thoracic radiotherapy is challenging, due to low proton density, large magnetic susceptibility differences between tissues and artefacts related to respiratory and cardiac motion (12, 48). The inability to optimise MR sequences within the MR-Linac workflow also precludes obtaining high image quality images in instances where sequences are inadequate but “locked down”. Hardware differences, e.g., B_0_ field strength, gradient specification, and RF coils, between standard diagnostic MR systems and MR-Linac systems, also affects image quality and the ability to acquire quantitative MR data. Both the ViewRay and Elekta systems permit diffusion-weighted imaging (DWI) to be acquired within the clinical workflow for certain treatment sites.

#### Electron Density Information

There is a lack of intrinsic electron density information associated with MRI. Ways of assigning CT density information to MR images include bulk density assignment, atlas-based methods or artificial intelligence approaches (56–58). The generation of a synthetic CT has been shown to work well in sites with tissue homogeneity such as prostate but its use in the thoracic region is more difficult (59). The current solution, used by the Elekta Unity system, is to use bulk density overrides of the OARs taking the mean electron density of each OAR from the CT.

#### Effect of the Magnetic Field

The effect of the magnetic field on dose distribution needs to be considered. The electron return effect (ERE) describes the effect of the magnetic field (Lorentz force) on secondary electrons (12, 48). The deposition of these secondary electrons at air-tissue interfaces can lead to increased doses. The ERE is reduced by modulating the treatment fields which is done as part of the Monaco plan optimisation (12). This is less of a concern with the MRIdian system due to its lower field strength (60).

#### Physiological Motion

The final challenge relates to the effects of cardiac and respiratory motion. The use of breath-hold imaging, respiratory gating, and 4D MRI are additional functions that would be beneficial in MRgRT for thoracic tumors (59, 61, 62). While both systems have the ability to monitor target movement (2-dimensionally) during treatment delivery, only the MRIdian can currently utilize real-time tumor imaging to modulate beam-on time during respiration. On the other hand, 4D MRI is not currently possible on either system. This may be less of a concern when 4D CT is used with initial planning for a single target such as SBRT, and especially if respiratory gating can be implemented with adaptive fractions (MRIdian only). However, in the absence of a complementary 4D CT and respiratory gating or the setting of multi-target treatment (as with locally advanced NSCLC), the lack of 4D MR imaging can pose a challenge.

An overview of the technical challenges related to MRgRT use in lung cancer has been summarized in **Table 2**, alongside their potential solutions (60, 62–64).

**Table 2:** Technical challenges and potential solutions associated with MRgRT in the thorax.

Challenge	Result	Potential solution/solution
Low proton density in lung tissue producing low MRI signal	Poor quality images resulting in difficulties with tumor and OAR delineation	Vendor provided optimised thoracic MR sequences, lower field strength, UTE sequences, hyper-polarized gas imaging or oxygen enhancement (10, 63, 64)
Respiratory and cardiac motion during image acquisition	Motion artefacts and larger planning margins	Breath hold imaging, 4D-MRI, gating or tracking (10, 62–64)
Susceptibility differences at air-tissue interfaces resulting in susceptibility induced field inhomogeneities	Reduced geometric accuracy and low signal	Lower field strength or FSE sequences (59)
Lack of intrinsic electron density information (including subsequent difficulty with synthetic CT generation)	Inaccurate electron density information leading to difficulties with dose calculation	Bulk density overrides from planning CT, research ongoing in specialized acquisition techniques, e.g., UTE sequence or the use of AI approaches (62)
Electron return effect (ERE)	Development of “hot spots” at air-tissue interfaces	Accounted for by planning algorithms or lower field strengths (60, 64)
Physiological motion during patient setup	Unrepresentative setup image	Acquire a new planning image
Physiological motion during treatment	Necessity for larger planning margins	Mid-position treatment, gating or tracking (64)

MRI, magnetic resonance imaging; OAR, organ at risk; FSE, fast spin echo; CT, computed tomography; UTE, ultra-short echo time; AI, artificial intelligence.

## Conclusion

This review presents the initial clinical experience of MRgRT in lung cancer. The potential benefits of MRgRT for lung cancer include improved target and OAR delineation and improved dosimetric accuracy. To unlock its full potential, we will still need to overcome some technical challenges, in particular the further optimisation of motion management.

To date, most of the clinical experience gained in the lung cancer setting has been with SBRT for stage I/II NSCLC or thoracic oligometastases from non-lung primaries, including (ultra-) central tumors. Overall, there appears to be a trend toward improved dosimetric accuracy with MRgRT, however, long-term clinical outcome data is awaited.

Ongoing clinical studies will focus on the feasibility of the definitive treatment of inoperable stage III NSCLC. In parallel, ongoing research into strategies aimed at overcoming the associated technical challenges will be required.

## Data Availability Statement

The original contributions presented in the study are included in the article/supplementary material. Further inquiries can be directed to the corresponding author.

## Author Contributions

CC wrote the first manuscript and sections from an Elekta perspective. PS read, reviewed and edited the first manuscript, and wrote sections relating to the ViewRay perspective. CC and PS contributed equally as first authors. DC, RC, and MD helped to design and adapted the structure of the paper from the start until the end of the writing process. DC, RC, MD, OG, SH, A-MS, MS, CR, GV, JV, and MW-W read, reviewed, edited, and wrote sections related to their areas of expertise. FM and CF-F read, reviewed, and edited the final version of the paper. GV and DC read, reviewed, edited throughout the whole writing process, and signed off the final paper. They both contributed equally to this work as last authors. All authors contributed to the article and approved the submitted version.

## Funding

Part of the publication fee has been funded by NIHR Manchester Biomedical Research Centre (Award number: BRC-1215-20007).

## Conflict of Interest

The University of Manchester, the Christie NHS Foundation Trust, University Medical Center Utrecht, and the Medical College of Wisconsin are members of the Elekta MR-Linac Consortium from which they have received financial and technical support under a research agreement with Elekta AB. The Christie NHS Foundation Trust is also supported by a Cancer Research UK Centres Network Accelerator Award Grant (A21993) to the ART-NET Consortium and RC is funded through ART-NET. Washington University in St. Louis has received research funding from Varian Medical Systems and Elekta AB. OG has received honoraria from ViewRay Inc. FM has received speaker fees from Elekta AB, is on the advisory board of Accuray and has received an MSD research grant. CF-F was supported by NIHR Manchester Biomedical Research Centre. CR is on the advisory board of Varian Medical Systems.

The remaining authors declare that the research was conducted in the absence of any commercial or financial relationships that could be construed as a potential conflict of interest.
